# Identifcation and fine mapping of *qHSW1*, a major QTL for hundred-seed weight in mungbean

**DOI:** 10.3389/fpls.2024.1510487

**Published:** 2025-01-24

**Authors:** Xuesong Han, Long Zhao, Juan Yu, Xingmin Wang, Shilong Zhang, Li Li, Changyan Liu

**Affiliations:** ^1^ Hubei Key Laboratory of Food Crop Germplasm and Genetic, Institute of Food Crops, Hubei Academy of Agricultural Sciences, Wuhan, China; ^2^ Key Laboratory for Crop Molecular, Breeding of Ministry of Agriculture and Rural Affairs, Wuhan, China; ^3^ Institute of Specialty Crops, Bijie Academy of Agricultural Sciences, Bijie, China

**Keywords:** fine mapping, mungbean, hundred-seed weight, QTL, RIL population, whole-genome resequencing

## Abstract

Mung bean, an important economic crop, is considered a crop with relatively high levels of plant protein constituents and is consumed as both a vegetable and a grain. Among various yield-related traits, hundred-seed weight (HSW) is crucial in determining mung bean production. This study employed a recombinant inbred line (RIL) population of 200 lines that were genotyped via whole-genome resequencing to exploit genetic potential in the identification of HSW-associated quantitative trait loci (QTLs) across four environments. We identified 5 QTLs for HSW, each explaining 2.46–26.15% of the phenotypic variance. Among these, *qHSW1* was mapped on chromosome 1 in all four environments, explaining 16.65-26.15% of the phenotypic variation. Fine mapping and map-based cloning procedures, along with progeny testing of recombinants, aided in narrowing the candidate interval for *qHSW1* to 506 kb. This identification of the *qHSW1* genomic interval and closely linked markers to *qHSW1* could prove valuable in breeding efforts for improved mung bean cultivars with higher seed weight.

## Introduction

Mung bean (*Vigna radiata* L.) is a warm-season legume crop from Asia. Owing to its exceptional nutritional value, short cropping cycle, and capacity to fix nitrogen, mung bean is extensively cultivated as a complete food source in the regions of South, East, and Southeast Asia (Graham and Vance, 2003). Hundred-seed weight (HSW) is an important yield-related trait in the mung bean industry. In general, consumers prefer large-seeded mung bean, and sprout producers require small-seeded mung bean. Therefore, the identification of major and/or stable quantitative trait loci (QTLs), as well as the development of molecular markers employed for marker-assisted selection (MAS) of HSW, is highly important for the genetic improvement of mung bean.

The QTLs controlling HSW have been identified via linkage mapping (Mei et al., 2009; Isemura et al., 2012; Kajonphol et al., 2012; Sompong et al., 2012; Muktadir et al., 2014; Somta et al., 2015) and genome-wide association studies (GWASs) in mung bean (Liu et al., 2022a, b; Han et al., 2022). Mei et al. (2009) utilized a recombinant inbred line (RIL) population derived from a cross between Berken (large-seeded) and ACC41 (small-seeded) and identified 11 QTLs for HSW across four environments, with individual QTLs explaining 2.6% to 15.1% of the phenotypic variation. Isemura et al. (2012) constructed a BC_1_F_1_ population consisting of 250 individuals from a cross between a wild relative and a local variety and developed the first genetic linkage map of mung bean with the number of linkage groups corresponding to the number of haploid chromosomes via 430 polymorphic molecular markers. They performed genetic mapping for HSW and located 7 QTLs, which were found in linkage groups 1, 2, 3, 7, 8, and 11, explaining 4.40% to 22.2% of the phenotypic variation. Kajonphol et al. (2012) used an F_2_ population consisting of 186 individuals from a cross between KUML29-1-3 and W021 for QTL mapping of agronomic traits and detected 6 QTLs for 100-seed weight located on linkage groups 2, 4, 8, 9, and 11, explaining 7.22% to 11.96% of the phenotypic variation. Sompong et al. (2012) used an F_2_ population from a cross between V1725BG and AusTRCF321925 and identified 5 QTLs for HSW located on linkage groups 1, 2, 8, 9, and 10, with individual QTLs contributing to phenotypic variation ranging from 6.83% to 18.41%. The QTL *SD100WT2.1* in linkage group 2 was located in the same region as the one identified by Kajonphol et al. (2012) et al., suggesting that they may represent the same locus. Muktadir et al. (2014) constructed an F_2:3_ population from the cross BARImung1 (small-seeded) and BARImung6 (large-seeded) populations and detected 4 QTLs for HSW in two environments, *qSDWT1.1*, *qSDWT8.1*, *qSDWT9.1*, and *qSDWT6.1*, located in linkage groups 1, 6, 8, and 9, with individual loci explaining 5.80% to 33.72% of the phenotypic variation. Among these, only *qSDWT6.1* was a novel locus, while the other three have been reported previously. Somta et al. (2015) detected 6 QTLs for HSW in an F_2_ population derived from KPS1 (large-seeded) and V718 (small-seeded) across three environments, with individual loci explaining 5.9% to 22.1% of the phenotypic variation. Rapid advancements in high-throughput sequencing technologies, coupled with the successful completion of the mung bean reference genome sequence by Kang et al. (2014), have made it feasible to detect genomic variation in an association mapping population. A recent study exploited the genetic potential of 217 mung bean accessions, landraces and cultivars from China through whole-genome resequencing and employed GWAS to identify 15 significantly associated SNPs (*P*< 0.001) with HSW out of 2,229,343 SNPs (Liu et al., 2022a). Similarly, another study exploited the genetic potential of 558 Chinese mung bean landraces by resequencing to identify 2,582,180 SNPs, followed by a GWAS to detect 25 SNPs significantly associated with HSW (Han et al., 2022).

Although many studies have reported QTLs for HSW and other yield-related traits in mung bean, only some of these were identified with major and stable effects across multiple environments (Liu et al., 2022a; Somta et al., 2015). Nevertheless, these QTLs often span broad genomic intervals due to limited sequencing resolution, low-density marker development, and insufficient recombinants within the candidate regions. The development of tightly linked markers is essential for mung bean MAS or marker-assisted breeding (MAB) programs. However, only a limited number of markers have been reported for HSW QTLs (Somta et al., 2022).

The genetic zygosity of the RIL population of 200 lines, developed through hybridization between parental lines (D2945 and D4702), was determined through genotyping on the basis of whole-genome resequencing and phenotypic performance in four distinct environments. High-resolution mapping procedures such as map-based cloning and progeny performance evaluation of recombinants for the HSW trait led to the identification and validation of a major, stable effect locus, *qHSW1*, in mung bean.

## Materials and methods

### Plant materials and phenotyping

The mung bean RIL population of 200 lines was developed through the single-seed descent method from a cross of D2945 (smaller seed size and lower HSW) and D4702 (larger seed size and higher HSW), two landraces that originated from the association population of 558 Chinese mung bean landraces reported previously (Han et al., 2022) and were obtained from the Hubei Provincial Medium-term Gene Bank for Crops (Wuhan, China).

Two hundred mung bean plants of the F_6_ generation, along with two parental lines, were evaluated across four locations from 2021-2022: Ledong (18.47° N, 108.54° E) in Hainan Province during 2021 (2021LD), Ezhou (30.40° N, 114.89° E) in Hubei Province during 2022 (2022EZ), Gucheng (32.29° N, 111.52° E) in Hubei Province during 2022 (2022GC) and Wuhan (30.58° N, 114.03° E) in Hubei Province during 2022 (2022WH). In 2021LD, mung bean lines were sown in late October and harvested in early January of the following year, benefitting from the tropical conditions of Ledong, Hainan Island. For 2022EZ, sowing occurred in mid-April during the spring season, with harvest occurring in early July. In both 2022GC and 2022WH, planting occurred in late June, with harvests conducted in October. Each location included two replicates. Every plot comprised a single 2-meter row with 11 plants spaced 20 cm apart within the row and 30 cm between rows. The weights of 100 randomly selected healthy, mature, and dry seeds were recorded via an electronic balance, and the average of three technical replicates was considered. The HSW used in subsequent analyses was derived from the average of these replicates per location.

### Resequencing, SNP identification, and genetic linkage map construction

For both the parental lines and the 200RILs, genomic DNA was extracted from young leaves of three two-week-old plants via the cetyl trimethyl ammonium bromide (CTAB) method, as described by Chen and Ronald (1999). A minimum of 2 µg of genomic DNA from each accession was used to construct sequencing libraries following the manufacturer’s protocol (Annoroad, Beijing, China). The libraries, with an average insert size of approximately 350 bp, were sequenced on a DNBSEQ-T7 platform, which produced 150 bp paired-end reads. The raw sequence data were cleaned via SOAPnuke v2.0.5 (Chen et al., 2018) to eliminate adaptor contamination and low-quality reads.

After performing the above filtering steps, good-quality reads were aligned to the high-quality chromosome-level mung bean M5311 reference genome assembly (Li et al., 2022a). For generating genomic alignments, BWA v0.7.17 (Li and Durbin, 2009) was employed, and the resulting SAM files were converted, sorted and indexed to the BAM format SAMtools v1.9 (Li et al., 2009). For SNP calling, GATK v4.1.8 (McKenna et al., 2010; parameters: –filter-expression QD< 2.0 || MQ< 40.0 || FS > 60.0 || SOR > 3.0 || MQRankSum< -12.5 || ReadPosRankSum< -8.0) was employed, and stringent filtering was applied to the SNPs on the basis of three criteria: (1) genetic diversity between the two parents; (2) support from more than four reads; and (3) conformity to a Mendelian segregation ratio of 1:1, as indicated by a *chi*-square test (*P*< 0.001). To facilitate mapping procedures, a genetic map was constructed with Joinmap v4.0 software by applying the *Kosambi* mapping function (Stam, 1993). A logarithm of odds (LOD) threshold of 5.0 was set.

### QTL analysis

QTL analysis was performed via the R/qtl package (Broman et al., 2003). The composite interval mapping (CIM) approach was employed to identify QTLs associated with HSW. The threshold LOD scores for each location’s HSW were determined through 1000 permutations (*P*< 0.05). QTL confidence intervals were determined via the 1.5 LOD-drop method. Additionally, a linear QTL model was applied to estimate the additive effects of the QTLs and the proportion of phenotypic variation explained.

### Marker development

Markers were developed in the *qHSW1* candidate interval via a penta-primer amplification refractory mutation system (PARMS) derived from whole-genome resequencing of the parental lines to further delineate and fine map the target genomic region. Each PARMS marker constituted a set of two forward primers and one reverse primer to efficiently determine genetic effects around the target locus. In addition, a FAM fluorophore tail sequence (5′-GAAGGTGACCAAGTTCATGCT-3′) or a HEX fluorophore tail sequence (5′-GAAGGTCGGAGTCAACGGATT-3′) was attached to the 5′ ends of both forward primers. These primers were synthesized by Gentides Biotech Co., Ltd. (Wuhan, China). The PCR plates were read via a TECAN Infinite M1000 plate reader, and SNP calling, as well as plot generation, was carried out via the online software SNpdecoder (http://www.snpway.com/snpdecoder/ accessed on 2 November 2022) with manual adjustments. PCR was performed following the protocol outlined by Li et al. (2022b). Detailed information about the PARMS markers is provided in **Supplementary Table S2**.

### Fine mapping of *qHSW1*

Using the high-density genetic map, a RIL designated D120, which harbored a heterozygous fragment in the *qHSW1* region within an otherwise homozygous background, was identified. To refine the mapping of *qHSW1*, plants carrying the heterozygous *qHSW1* region, selected from the selfed progeny of D120, were further self-pollinated to establish a large F_2_ population. Recombinant individuals from this F_2_ population were identified via PARMS markers flanking *qHSW1*. Fine mapping was performed via a progeny-testing approach, as described by Han et al. (2020), in which the selected recombinant plants were selfed to produce homozygous recombinant plants (HRs) and nonrecombinant plants (HNRs) through marker-assisted selection. Significant phenotypic differences (P< 0.01) between HRs and HNRs indicated that the recombinant plant carried *qHSW1* within its heterozygous region. Through progeny performance evaluation of all the recombinants, the *qHSW1* locus was further delineated to a further narrowed interval.

### Statistical analyses

Descriptive statistics and analysis of variance (ANOVA) for the HSW were estimated across the four locations via IBM SPSS Statistics v29.0.0. The broad-sense heritability (*h^2^*) was determined according to the method described earlier by Hallauer and Miranda (1998), as described below:


$$h^{2}\;=\frac{\sigma_{\text{g}}^{2}}{\sigma_{\text{g}}^{2}+{\text{σ}}_{\text{ge}}^{2}/\text{n }+\sigma_{\text{ϵ}}^{2}/\text{rn}}$$

where $\sigma_{ɡ}^{2}$ represents the genetic variance, $\sigma_{ɡe}^{2}$ represents the interaction of genotype with the environment, $\sigma_{\epsilon }^{2}$ denotes the error variance, n represents the number of environments with replications, and r represents the number of replications per environment. The values for $\;\sigma_{ɡ}^{2}$ , ${\text{σ}}_{\text{ge}}^{2}$ , and $\sigma_{\text{ϵ}}^{2}$ were obtained from variance components via a generalized linear model (GLM). Correlation analysis was performed via Origin 2021 software. Differences between groups were evaluated with a two-tailed Student’s *t* test.

## Results

### Phenotype analyses

**Figure 1:**
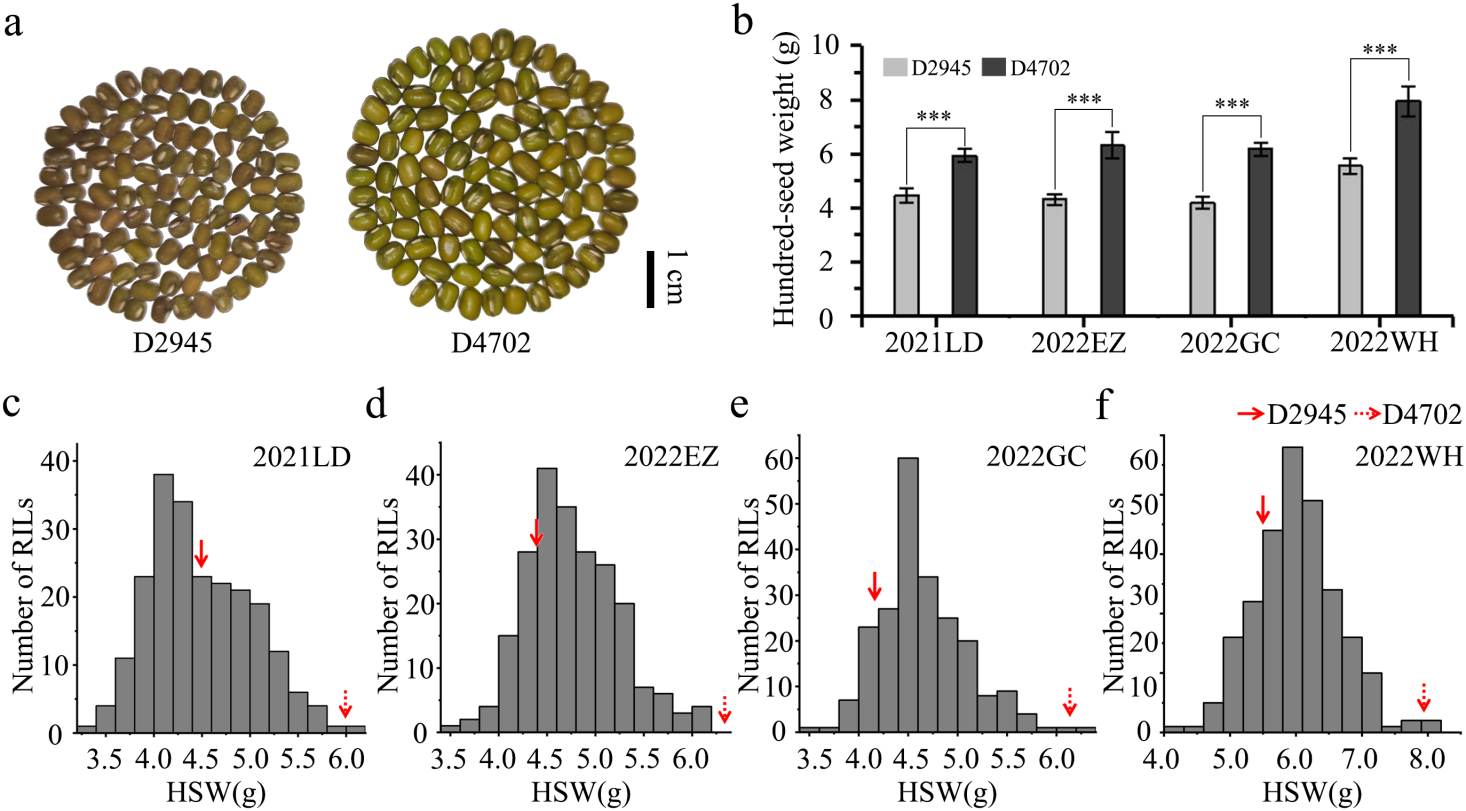
Phenotypic performance evaluation of two parental lines and 200 RILs. **(A)** Seed morphology of D2945 and D4702. The scale bar is equivalent to 1 cm. **(B)** Significant differences were observed for the HSW trait between D2945 and D4702 across four different environments (2021LD, 2022EZ, 2022GC, 2022WH). *** denotes significance at 0.001. **(C-F)** Plots showing frequency distributions of the D2945×D4702 RIL population for HSW in four different environments. 2021LD **(C)**, 2022EZ **(D)**, 2022GC **(E)**, and 2022WH **(F)**. The red arrows indicate values of the two parent lines.

The seed morphology and HSW in all four environments (2021LD, 2022EZ, 2022GC, and 2022WH) differed significantly between the two parental mung bean accessions D2945 and D4702 (**Figures 1A, B**). The HSW in the D2945×D4702 RIL population displayed a continuous and approximately normal distribution pattern (**Figures 1C-F**, **Table 1**), indicating that HSW was a quantitatively inherited trait in this study. The overall broad-sense heritability for the HSW trait was 0.83 across the four environments. Significant correlation values ranging from 0.65 to 0.83 (**Table 2**) were also observed. Additionally, genotype, environment, and their interaction effects were found to be significant in exhibiting the HSW trait within the RIL population (**Table 3**). These findings imply that genetic variation predominantly governs the phenotypic diversity of HSW.

**Table 1:** Phenotypic performance of the HSW in RIL population under four environments.

Environments	Parents (g)		RIL families (g)					
	D2945	D4702	Means	SD	Minimum	Maximum	Skewness	Kurtosis
2021LD	4.46	5.93	4.48	0.53	3.28	6.00	0.39	-0.37
2022EZ	4.32	6.31	4.75	0.48	3.57	6.19	0.53	0.20
2022GC	4.18	6.17	4.65	0.44	3.45	6.20	0.67	0.70
2022WH	5.55	7.95	6.01	0.64	4.29	8.09	0.36	0.40

**Table 2:** Pearson correlation coefficients (*r*) for HSW among environments.

Environments	2021LD	2022EZ	2022GC
2022EZ	0.68***	1	
2022GC	0.73***	0.83***	1
2022WH	0.65***	0.82***	0.82***

***Significant at the 0.001 level.

**Table 3:** Analysis of variance for HSW in the D2945×D4702 RIL population across four environments.

Source of variation	Type III SS	DF	MS	*F*	Significance level
Genotype	348.63	221	1.58	25.38	<0.001
Environment	585.93	3	195.31	3142.07	<0.001
Genotype×Environment	88.45	658	0.13	2.16	<0.001
Error	45.87	738	0.06		
Total	1068.88	1620			

SS, Sum of Squares. DF, Degree of Freedom. MS, Mean Square.

### SNP detection and genetic linkage map construction

A total of 1,673,185,938 clean reads were obtained from whole-genome resequencing in this study. Among them, the numbers of reads for D2945, D4702, and RILs were 83,967,545, 59,874,725, and 1,529,343,668, respectively. The sequence depths for D2945 and D4702 were 26.16× and 18.65×, respectively, whereas the average sequence depth for the RILs was 2.38×. Detailed sequencing data for the two parental accessions and RILs used in this study can be found in **Supplementary Table S1**.

**Figure 2:**
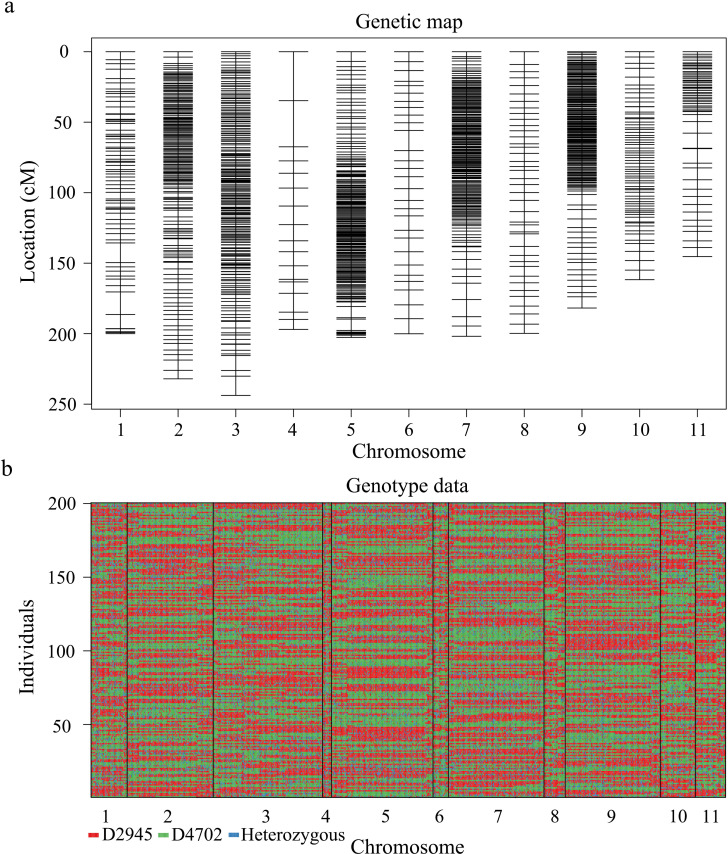
High-resolution genetic linkage map and genotyping map of the D2945×D4702 RIL population generated via whole-genome resequencing. **(A)** Distribution and genetic location of SNP markers on 11 mung bean chromosomes. The black bar indicates an SNP marker. **(B)** The graphic genotype of 200 RILs. Red, D2945 genotype; green, D4702 genotype; blue, heterozygous genotype.

SNP identification yielded 889,575 SNPs between the two parental accessions. After filtering, 3521 high-quality SNPs were utilized to construct a linkage map. Finally, a linkage map comprising 1194 SNPs randomly distributed across 11 linkage groups was developed via the *Kosambi* mapping function integrated in Joinmap v4.1 software (**Figures 2A, B**), with an overall length of 2,493.82 cM and an overall marker-to-marker distance of 2.09 cM (**Table 4**).

**Table 4:** Description of characteristics of the genetic linkage map.

Chromosome	Nomber of SNPs	Map length (cM)	Average interval (cM)
1	67	331.04	4.94
2	162	232.04	1.43
3	206	243.82	1.18
4	17	197.01	11.59
5	191	297.23	1.56
6	29	276.11	9.52
7	180	227.86	1.27
8	40	199.82	5.00
9	179	181.82	1.02
10	66	161.72	2.45
11	57	145.35	2.55
Total	1194	2493.82	2.09

### QTL mapping

**Figure 3:**
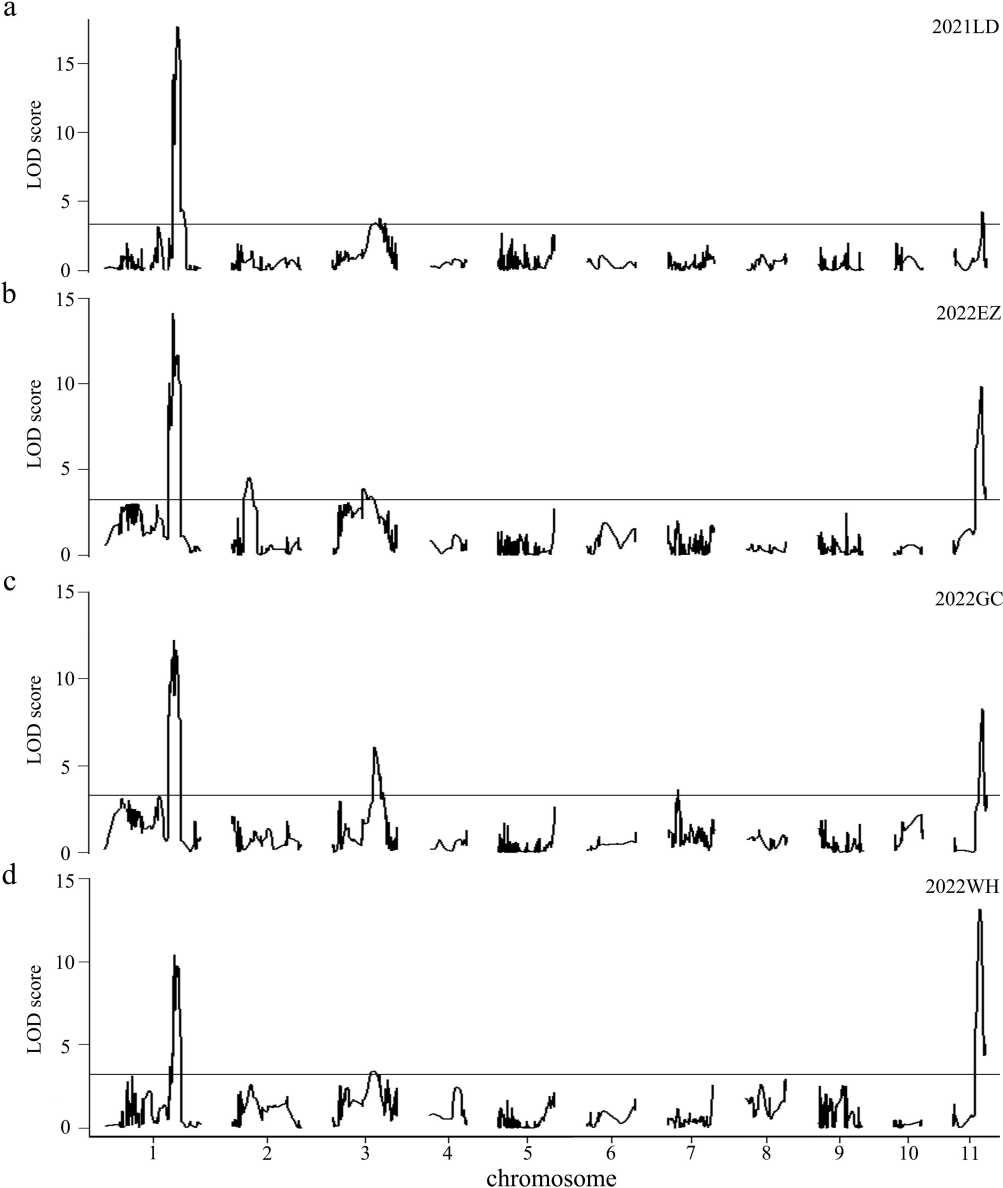
Putative QTLs for HSW were detected in 200 RILs across four distinct environments. The environments are represented as follows: **(A)** 2021LD for 2021 Ledong, **(B)** 2022EZ for 2022 Ezhou, **(C)** 2022GC for 2022 Gucheng, and **(D)** 2022WH for 2022 Wuhan. The LOD values are shown, with horizontal lines indicating the LOD thresholds determined from 1000 permutation tests at a significance level of 0.05, which were calculated via the CIM model in R/qtl software.

Combining the genotypes and phenotypes of the RILs, QTL mapping was performed through the CIM function in R/qtl, with the LOD threshold determined as 3.25 through a permutation test consisting of 1,000 iterations (*P*< 0.05). A total of 5 QTLs for HSW were identified through single-environment QTL analysis; these QTLs were located on chromosomes 1, 2, 3, 7 and 11 (**Figure 3**, **Table 5**), with LOD values ranging from 3.41 to 17.66, and explained 2.46% to 26.15% of the phenotypic variation (R^2^). The positive alleles of these five QTLs were all from the high-HSW accession D4702. Among them, *qHSW1, qHSW3* and *qHSW11* were detected in all four environments, and *qHSW2* and *qHSW7* were environment specific. Moreover, *qHSW1* and *qHSW11* explained more than 10% of the observed phenotypic variation (**Table 5**). These findings suggest that *qHSW1* and *qHSW11* are stable major QTLs. The *qHSW1* has shown significant effects in this and previous studies, indicating its potential importance in trait variation. Thus, we choose the *qHSW1* for fine mapping.

**Table 5:** Putative QTLs detected for mungbean HSW in RIL families across four environments.

QTL	Env.^a^	Chr.^b^	Marker Interval	LOD	Additive^c^	Dominant^d^	R^2^(%)^e^
*qHSW1*	2021LD	1	chr1:53604054- chr1:54381462	17.66	0.35	-0.07	26.15
	2022EZ	1		14.13	0.21	-0.09	16.65
	2022GC	1		12.22	0.19	-0.20	17.43
	2022WH	1		10.44	0.30	-0.21	23.37
*qHSW2*	2022EZ	2	chr2:11079302- chr2:19598200	4.52	0.14	-0.02	6.00
*qHSW3*	2021LD	3	chr3:11976317- chr3:13152588	3.81	0.08	-0.21	2.46
	2022EZ	3		3.90	0.11	-0.26	5.65
	2022GC	3		6.09	0.14	-0.23	8.84
	2022WH	3		3.41	0.12	-0.10	3.51
*qHSW7*	2022GC	7	chr7:6659004- chr7:9992799	3.62	0.03	-0.42	3.75
*qHSW11*	2021LD	11	chr11:21257672-chr11:22193570	4.25	0.10	-0.08	13.10
	2022EZ	11		9.87	0.19	-0.07	13.54
	2022GC	11		8.27	0.16	-0.15	12.18
	2022WH	11		13.18	0.23	-0.05	13.43

a, Env., environment; 2021LD, 2022EZ, 2022GC, and 2022WH represent the environments 2021Ledong, 2022Ezhou, 2022Gucheng and 2022Wuhan, respectively; b, chromosome; c, additive effect; d, dominant effect; e, variance explained by the QTL.

### Fine mapping of *qHSW1*

**Figure 4:**
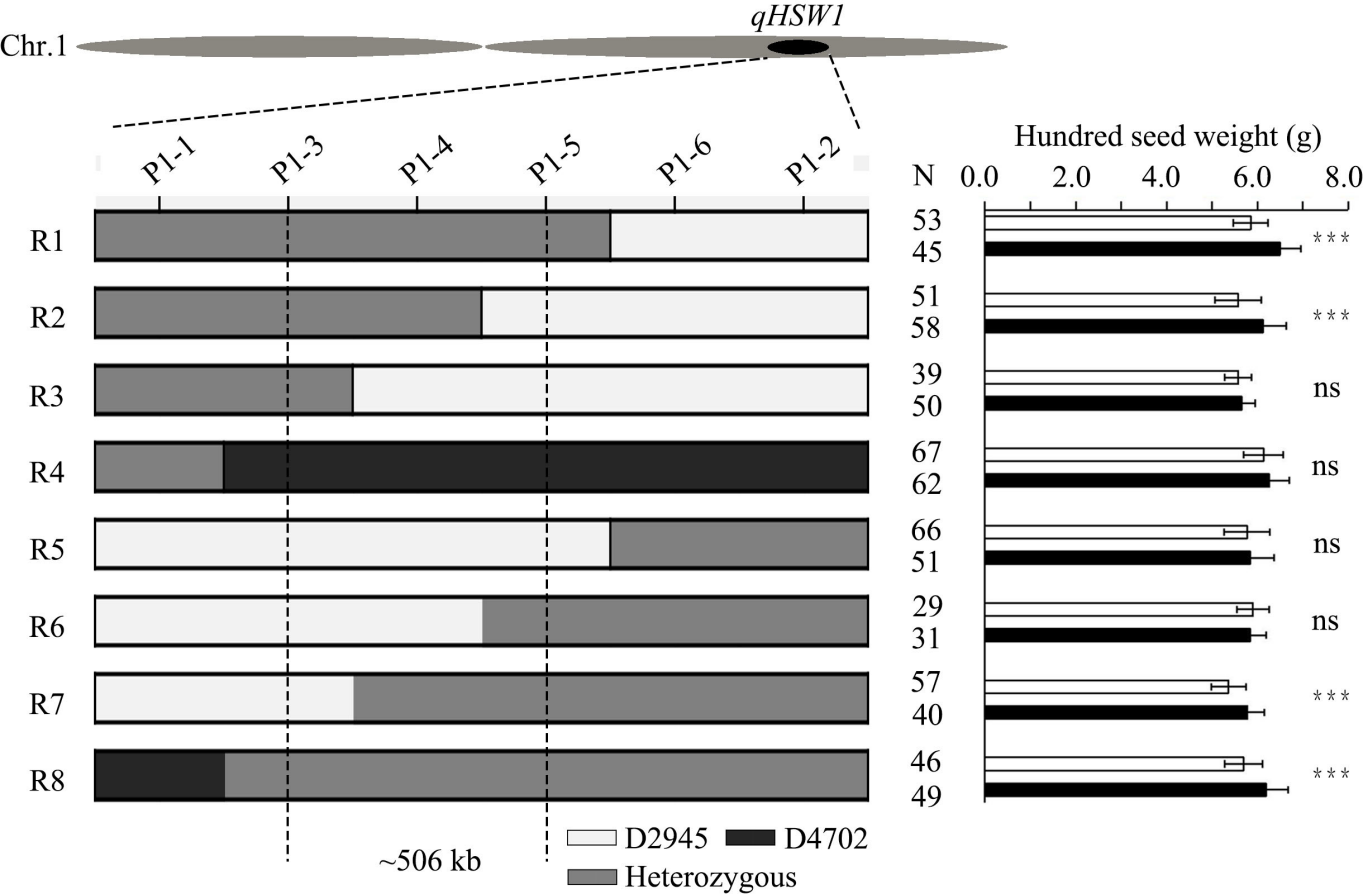
Fine mapping of *qHSW1* and the structure of the candidate gene. Graphical genotypes of eight recombinant lines and progeny test results for each recombinant line. The gray boxes represent heterozygous genotypes with D2945/D4702 chromosome segments, whereas the white and black boxes represent homozygous D2945 and D4702 segments, respectively. The significance was estimated by Student’s *t* test. ***Significant at the 0.001 level. N, sample size. ns, not significant.

For fine mapping of *HSW1*, a RIL, named D120, which was heterozygous in the *qHSW1* region with a homozygous background, was screened out. Twenty plants with a heterozygous region of *qHSW1* that were selected from the selfing progeny of D120 were self-pollinated to develop a large F_2_ population. All the seeds of the large F_2_ population were planted in the field, which resulted in 3,715 plants. A total of 34 recombinants were identified from this population via the PARMS markers M1-1 and M1-2 flanking the *qHSW1* region. These 34 recombinants were further classified into eight distinct crossover events via four newly developed PARMS markers (M1-3 to M1-6) within the M1-1 to M1-2 region. Eight recombinants were selected from the 34 recombinant plants to represent these eight distinct crossover events. Genotype identification and phenotypic difference analysis were subsequently conducted on homozygous recombinant plants and homozygous nonrecombinant plants selected from the selfing progeny of each of the eight recombinants. Progeny performance evaluation revealed significant differences in HSW between homozygous recombinant plants and nonrecombinant plants corresponding to the four recombinants (R1, R2, R7, R8) with the heterozygous *qHSW1* genotype in the M1-3 to M1-5 region. However, no significant difference in HSW was detected between the homozygous recombinant plants and the homozygous nonrecombinant plants corresponding to the four recombinants (R3, R4, R5, R6) with the homozygous *qHSW1* genotype in the M1-3 to M1-5 region. These findings indicated that *qHSW1* is located within the approximately 506 kb (Chr1:53868960~54374894 bp in M5311 RefGen) chromosomal region between M1-3 and M1-5 (**Figure 4**).

### Genetic effects and potential value of *qHSW1* in mung bean breeding

To estimate the genetic effect of *qHSW1*, a set of NILs, designated *qHSW1* ^C2945^ and *qHSW1* ^C4702^, were generated by selfing D120 with genotype selection. The investigation and analysis of important agronomic traits in *qHSW1* ^C2945^ and *qHSW1* ^C4702^ revealed significant differences in HSW between NILs. The HSW of *qHSW1* ^C2945^ was approximately 0.54 g lower than that of *qHSW1* ^C4702^, and in addition, the number of seeds per pod of *qHSW1* ^C2945^ was significantly (*P<* 0.05) greater than that of *qHSW1* ^C4702^ (**Table 6**). No significant differences were observed in traits such as pod width, pod length, protein contentof seed, starch content of seed, flowering time, plant height, or branch number (**Table 6**).

**Table 6:** Phenotypes of agriculturally important traits of *qHSW1* NILs.

Trait	*qHSW1* ^D2945^	*qHSW1* ^D4702^	*P*-vaule	N^a^
100-seed weight(g)	5.73	6.27	7.62E-04	29/31
Seeds per pod	10.86	10.36	0.02	29/31
Pod width(mm)	5.00	5.12	0.05	29/31
Pod length(mm)	87.44	88.99	0.28	29/31
Protein content of seed (%)	25.13	25.14	0.98	29/31
Starch content of seed (%)	49.41	50.64	0.05	29/31
Days to flowering time (days)	40.09	40.95	0.16	29/29
Plant height(cm)	59.83	60.2	0.58	29/31
Branch number	1.54	1.59	0.31	29/31

a, samples size, *qHSW1* ^D2945^/*qHSW1* ^D4702^ homozygotes.

**Figure 5:**
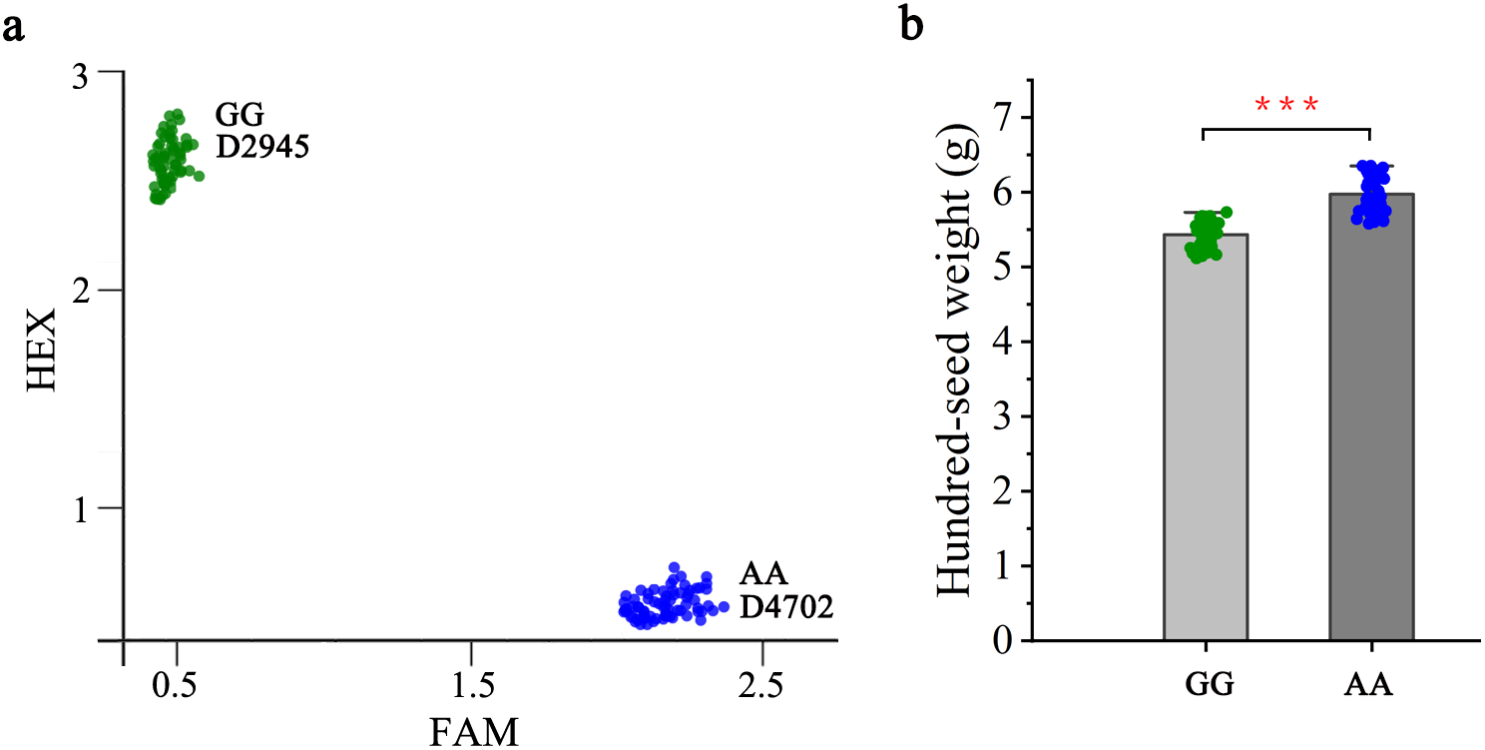
Genetic effect of *qHSW1* on hundred-seed weight in the D2945×D4702 RIL population. **(A)** Genotyping results for the 94 lines randomly selected via markers M1-3. The green, red, and blue dots represent the D2945 allele and D4702 allele, respectively. **(B)** Comparisons of HSW between lines with contrasting genotypes. ***, significant at *P*<0.001.

To evaluate the potential utility of *qHSW1* in mung bean breeding, marker M1-3, which were successfully classified into two distinct groups, was used to exploit allelic variations present within the D2945×D4702-derived RIL population. For validation, a subset from the RIL population (approximately 94 lines) was randomly selected for genotyping with the M1-3 marker. Among these, 50 lines presented the same genotype as D2945, whereas 40 lines presented the same genotype as D4702 (**Figure 5A**). Association analyses revealed that the M1-3 genotype was significantly associated with HSW (*P<* 0.05) in the D2945×D4702 RILs, where the D4702 allele had predominantly higher HSW (mean 5.97 g) contributions than the D2945 allele (5.43 g) (**Figure 5B**). Thus, marker M1-3 can be effectively employed in marker-assisted selection programs for HSW in mung bean.

## Discussion

### Comparisons of *qHSW1* with previously reported QTLs for HSW

Many of these loci had minor effects and were mapped to wider genomic/physical intervals (Somta et al., 2022). Enhancing the mapping resolution necessitates enormous population sizes and a higher density pool of good-quality markers. Furthermore, maintaining adequate replications while minimizing phenotyping errors, along with a high-resolution genetic map, would increase the precision of QTL mapping procedures. Here, we employed 200 mung bean RILs and developed a genetic map based on high-quality SNP markers that were genotyped via a whole-genome resequencing strategy. Two major QTLs, *qHSW1* and *qHSW11*, were consistently identified for HSW across four environments. Further literature analysis revealed that *qHSW1* corresponds to the QTL *Sd100w8.1.1* described by Isemura et al. (2012) and aligns with the QTL *Sd100wt8.1* identified by Kajonphol et al. (2012). Additionally, it partially overlaps with the QTL *qSDWT8.1* reported by Muktadir et al. (2014). Collectively, these findings indicate that *qHSW1* is likely a significant contributor to the natural variation observed in HSW within mung bean germplasm.

### Implications of *qHSW1* in mung bean molecular breeding programs

Marker-assisted selection employs molecular markers that are intimately or tightly linked to the target traits, thus offering opportunities for the selection of plants possessing favorable alleles that could significantly impact the targeted traits (He et al., 2014). Marker-assisted selection has been extensively applied in multiple economic crops, such as rice (Gouda et al., 2020), maize (Xu et al., 2020) and wheat (Sun et al., 2020), but rarely in mung bean. Owing to the limited advancements in functional genomics research on mung bean, only a small number of closely linked, reliable and MAB-compatible markers related to phenotypic traits have been identified (Somta et al., 2022). Consequently, there are few reports of MAS in mung bean.

In conclusion, *qHSW1* exhibited stable genetic effects across four different environments over multiple generations. The major effect of the HSW QTL *qHSW1* identified in this study was able to explain ~21% of the phenotypic variation and varied in HSW by 0.54 g (**Tables 5**, **6**); thus, introgression of this QTL is an efficient approach for mung bean HSW improvement. This QTL has no additional influence on pod width, the protein content of seeds, flowering time, plant height, or branch number; and represents easy-to-use and specific features for MAS. Through a progeny test strategy, we successfully narrowed *qHSW1* down to a 506 kb region. The M1-3 marker was tightly linked to *qHSW1*, thus allowing deciphering of exact *qHSW1* coordinates and exclusively introgressing the candidate locus to develop an ideotype mung bean accession. As a next step, further fine mapping of *qHSW1* will be performed, and functional markers for the candidate gene for *qHSW1* will be developed.

## Data Availability

The datasets presented in this study can be found in online repositories. The names of the repository/repositories and accession number(s) can be found below: https://www.ncbi.nlm.nih.gov/, PRJNA1034219.
